# The perception and association between depression and academic stress among female undergraduate nursing students: a cross-sectional study

**DOI:** 10.3389/fpubh.2024.1414469

**Published:** 2024-06-18

**Authors:** Shaherah Yousef Andargeery

**Affiliations:** Nursing Management and Education Department, College of Nursing, Princess Nourah bint Abdulrahman University, Riyadh, Saudi Arabia

**Keywords:** depression, depressive symptoms, academic stress, nursing students, academic setting

## Abstract

**Introduction:**

Academic demands and stressors put nursing students at increasing risk of depression. The aims of the study examine the perceived level of depression and academic stress and investigate the association between these variables among nursing students in Saudi Arabia.

**Methods:**

A cross-sectional design was used in this study. Data was collected using depression subscale from DASS-21 scale and the Academic Stress Inventory scale.

**Results:**

A total of 237 students participated in the study. Nursing students perceived normal to mild levels of depression. Students perceived moderate levels of academic stress in all subscales, except for time management and test stress which were high. All academic stress subscales had a positive statistically significant correlation with depression. The regression model explains 49.0% of the variance in the depression scale and indicated that the main significant predictor of depression is studying in group stress, followed by self-inflected stress, study year, and sleep problem. The mean score of depression for first year students was significantly different than students in other study years.

**Discussion:**

Nurse educators should facilitate group formation and teach students about time-management, problem-solving, negotiation, and coping strategies to deal with academic expectations and demanding situations. Understanding the causes, limiting the exposure to negative influences, and seeking support as needed are important. To enhance the quality of sleep, students should maintain a consistent sleep schedule, while avoiding stimulating activities before bedtime. Future research should focus on a longitudinal study and other variables such as quality of life, satisfaction with the quality of teaching, and support from others.

## 1 Introduction

Attending university can be an exciting and transformative experience, yet the education itself can be a very stressful experience (1). Factors such as academic workload, transition and adjustment to a new environment, building new social networks, and lack of support (2–4). Education is frequently seen as a path to future opportunities and career expectations. The pressure to make the right decision regarding the selection of the field of the study and career paths can evoke stress and anxiety about the future (5). These factors may contribute to the development or exacerbation of depression in some students (2–5).

Depression is the most common health issue that confront students during their education (6). The prevalence of psychiatric disorders can be higher among students four times than general population (7). High depression rate was identified in previous studies with a prevalence of 30.2 to 38.7% among university students (6, 8).

Nursing students, among the other fields of medical sciences, are exposed to more stressors while studying and they are at high risk of depression more than any other students in non-medical science fields (6) Depression is associated with disruption of biological rhythms, fatigue, and insomnia (9). Good mental health is essential for students’ academic performance, concentration, self-efficacy, and quality of life (10). According to Kwak et al. (11), prolonged academic stress can significantly impact students’ mental health and is consequently leading to depression. In their findings, they found a significant positive correlation between academic stress and depression (*r* = 0.36, *p* < 0.001). Their results also revealed that academic stress was the strongest predictor affected the depression of nursing students.

Zhang et al. (12) indicated that high levels of stress can potentially cause negative mental health outcomes and unhealthy behaviors among college students. They also indicated that poor sleep quality significantly enhances the perceived stress of nursing students, which in turn influences depression and anxiety symptoms. Another study implemented by Hsieh et al. (13) revealed that 46% of the variance of stress, burnout, and quality of sleep was explained by depressive symptoms among psychiatric nurses. They also explained that there was indirect association between stress and depressive symptoms that was mediated by occupational burnout and quality of sleep (12). They also explained that depressive symptoms can be improved by the improvement of quality of sleep and the reduction of occupational burnout.

Students who are depressed are prone to have high absenteeism from lectures and low cumulative grade point averages (GPA) than the students who do not have depression (14). Similarly, the academic demands and pressure put nursing students at increasing risk of depression and anxiety, which could consequently affect self-efficacy, interpersonal relationships with parents and friends, and professional life’s aspects of these students (15–20).

Nursing students are the future of nursing workforce, while nursing education is the vital place to mitigate the impact of psychological health and factors that influence nursing students’ academic performance (3, 21, 22). Students pass through a transition phase as they move from high school to enter university. Students become responsible for acquiring knowledge and skills necessary for future professional performance. During this transition phase, nursing students become responsible for their own decisions and lifestyles, start to cope with a new educational environment, and adapt with academic, social, and professional training demands (23).

Academic stress refers to the psychological and emotional pressure faced by students who are pursuing their education due to the increased academic-related demands that exceed students’ ability to cope with the pressures (24). It is a rapidly growing phenomenon that influences students globally (25). Evidence showed that first year nursing students had higher depression rate than fourth year nursing students and by the time they start clinical practice (26). Previous literature showed that a high level of stress in nursing students was associated with depression and anxiety (9, 27–29). The authors reported that the causes of stressors include unfamiliarity with academic environment, concerns over failure, performance evaluation by the instructors, and ineffective communication with instructors. The other stressors in the educational settings are high academic expectation including course work assignments, intensive study workload, information overload, increase competitiveness, lack of emotional support, inability to balance between academic and social life, and having clinical practicum and skills examinations (19, 27, 32, 33).

The authors identified other sources of stress that were reported by Pakistani students which were language difficulty and irrelevant field selection (30). Family distancing, lack of leisure time and coping strategies, lifestyle and dietary changes, and inadequate motivation can lead to high physical and emotional disturbances including anxiety, stress, and depression (31, 32). The common stressors identified during professional training were related to seeing patients suffering, fear of making mistakes or causing harm or death when providing care to patients, and miscommunication with patients, physicians, and other healthcare providers (33). Lack of clinical experience, feeling incompetent, or inadequate nursing knowledge and skills to perform clinical examination, such as giving injection were other professional training stressors identified by nursing students. If these academic stressors were not resolved, students became prone to high levels of depressive symptoms. Early identification of depression is critical to limit the negative consequences of its’ triggers.

To the best of the author’s knowledge, there is a paucity of evidence that explain the prevalence of depression and academic stress among female nursing students who are considered in the literature as at a higher risk of acquiring psychological disorders, particularly in Saudi Arabia. The studies found in Saudi Arabia were conducted during COVID-19, which is considered as a covariate. Continuous evaluation of the students will allow prompt management of depression and stress. Therefore, the aims of the study are to: (1) Examine the perceived level of depression and academic stress; and (2) Examine the association between academic stress and depression among nursing students in Saudi Arabia.

## 2 Materials and methods

### 2.1 Study design

Cross-sectional study design was used to examine the perceived level of depression and academic stress and their association among nursing students.

### 2.2 Setting

The study was implemented in the College of Nursing at one of the biggest universities in Riyadh, Saudi Arabia.

### 2.3 Sampling

A Purposive sampling method was used in this study. Cohen (34) strategy was used to determine the required sample size. All participants have an equal opportunity to participate in this study if they met all the following criteria: (1) their age is 18 years or older; (2) nursing students who were enrolled in College of Nursing; and (3) has spent at least 6 weeks in the nursing program. The participants were excluded from the study if they were studying in the master’s and PhD programs. Two-tailed tests with a medium effect size of 0.15 and a statistical power analysis of 0.80 were used. To compensate for the missing data and to pretest the questionnaires, 40% was added to the required sample size. The minimum sample size of 201 students needed to have a confidence level of 95% with a margin error of 5%.

### 2.4 Measurement

#### 2.4.1 Demographic data

Demographic questions include age, GPA, study year, place of residence, and sleep problem.

#### 2.4.2 Depression measurement

The participants were asked to fill out the depression subscale using Depression, Anxiety, Stress Scale (DASS-21), which was developed by researchers at the University of New South Wales (35). The scale was validated in previous studies that were implemented in Saudi Arabia and reported a Cronbach’s alpha reliability between 0.89 and 0.94 (36, 37). In this study, the Cronbach’s alpha coefficient reliability for the depression subscale is 0.904, suggesting that the items have a relatively high internal consistency.

The depression subscale consists of 7 items, and it scored in a 4-point Likert-type scale. The participants were asked to rate each item from 0 to 3; with (0) score indicating “did not apply to me at all” and a score of (3) indicating “applied to me very much or most of the time.” The scores range between 0 to 4 indicate normal depression level. The scores range from 5 to 6 indicate mild depression level. The scores range between 7 to 10 indicate moderate level of depression. Scores range from 11 to 13 indicate severe level of depression. Scores range from 14 to 21 indicate extremely severe depression (35, 38). The mean score was calculated for the depression subscale with the higher scores indicate extremely severe symptoms of the domain.

#### 2.4.3 Academic stress measurement

The participants were also asked to fill out the Academic Stress Inventory (ASI) scale which measures academic stress. The ASI scale was developed by Lin and Chen (39) and the scale was confirmed to the reliability and validity requirements. Item analyses and content validity after drafting pre-test questionnaires were applied by panel of experts to ensure validity. The accumulated total variance explained was 70.91%. The reliability test reported an alpha value between 0.85 and 0.92 for the subscales (39). In this study, English version was used, and the content validity index was 0.93. The Cronbach’s alpha coefficient reliability for the ASI subscales was between 0.704 and 0.897 indicating that the items have good to high internal consistency in the current study.

ASI scale consists of seven subscales. Teacher stress consists of 9 items and includes questions related to teaching, teaching materials, and exercise items. Results stress consists of 5 items and includes questions about stress from parents’ expectations and conflicts between opinions and expectations of grades. Test stress consists of 4 items and includes questions related to concern about tests’ preparations and remake the compulsory courses. Studying in groups stress consists of 5 items and includes questions related to exercise reports and grouping. Peer stress consists of 4 items and includes questions related to academic competition and peer interferences. Time management stress consists of 3 items and includes questions related to time management, social activities, and student association. The last subscale is self-inflicted stress which consists of 4 items and includes questions related to self-expectation, and interests of course selection.

This scale is made up of 34 items. The ASI items are scored on a 5-point Likert-type scale and the scores range from 1 to 5; A score of 1 was coded as completely disagree while a score of 5 was coded as completely agree. The scores for each subscale were added and the averages were calculated. A higher score in each factor indicates a high degree of stress produced by this factor.

### 2.5 Ethical approval

Institutional Review Board (IRB) approval was received before conducting the study, approval (No. 22–0488). The potential participants were informed that participation in the study will be voluntary, the survey will be anonymous, and confidentiality will be emphasized. They were assured that the collected data does not include any identifier. The participants were assured that they would choose not to answer some or all questions or withdraw from the study at any time without penalty. Potential participants who have not given their agreement on the consent form were prevented from proceeding with the survey.

### 2.6 Recruitment and data collection procedure

An invitation letter was sent by the Nursing Students Club to potential participants through email. An online survey with written instructions was sent to the respondents who agreed to participate. The data was collected from January 2023 and March 2023.

### 2.7 Data analysis

Data was analyzed using SPSS version 27. Descriptive statistics were used to analyze descriptive data. Post-hoc analysis was conducted to examine the difference in the mean scores of depression between study years. Pearson correlation coefficient was used to test the direction of association between the academic stress and depression subscales. Multiple regression model was implemented using the demographic variables and the academic stress subscales as predictors of depression. The statistical significance level was defined as *p* < 0.05 and confidence intervals of 95%.

## 3 Results

The total number of participants was 237 nursing students. As depicted in Table 1, the mean age of nursing students is 19.78 (SD = 1.43) with the mean GPA of 3.94 (SD = 0.73). The majority of the participants were studying in the first year (35.9%), reside with their families (91.1%), and did not have sleep problems (53.2%).

**Table 1:** Sample Characteristics (*n* = 237).

	***M* (SD)**	***n* (%)**
**Age**	19.78 (1.43)	
**GPA**	3.94 (0.73)	
**Study year**		
1st		85 (35.9)
2nd		55 (23.2)
3rd		54 (22.8)
4th		43 (18.1)
**Residence**		
With family		219 (92.4)
Dormitory		18 (7.6)
**Sleep problem**		
Yes		111 (46.8)
No		126 (53.2)

*M*, Mean.

SD, Standard deviation.

*n*, number of participants.

Table 2 shows that most of the students perceived normal to mild levels of depression (57.4%) with the mean score of 5.40 (SD = 2.72), yet the rest (42.6%) perceived moderate to extremely severe levels of depressive symptoms. Table 3 illustrates that students reported moderate levels of teachers’ stress (*M* = 29.09, SD = 8.08), results stress (*M* = 14.15, SD = 5.18), studying in group stress (*M* = 14.06, SD = 5.13), peer stress (*M* = 10.22, SD = 3.86), and self-inflicted stress (*M* = 12.23, SD = 4.22). However, students perceived high levels of test stress (*M* = 13.63, SD = 4.55), and time-management stress (*M* = 9.17, SD = 3.58).

**Table 2:** Depression levels (*n* = 237).

**Study variable**	***M* (SD)**	**Normal** *n* (%)	**Mild** *n* (%)	**Moderate** *n* (%)	**Severe** *n* (%)	**Extremely Severe** *n* (%)
Depression	5.40 (2.73)	101 (42.6)	35 (14.8)	50 (21.1)	22 (9.3)	29 (12.2)

**Table 3:** Academic stress inventory levels (*n* = 237).

Study variables	*M* (SD)	Low *n* (%)	Moderate *n* (%)	High *n* (%)
Teachers’ stress	29.09 (8.08)	34 (14.3)	133 (56.1)	70 (29.5)
Results stress	14.15 (5.18)	77 (32.5)	122 (51.5)	38 (16.0)
Test stress	13.63 (4.55)	36 (15.2)	92 (38.8)	109 (46.0)
Studying in group stress	14.06 (5.13)	79 (33.3)	118 (49.8)	40 (16.9)
Peer stress	10.22 (3.86)	83 (35.0)	123 (51.9)	31 (13.1)
Time-management stress	9.17 (3.58)	63 (26.6)	80 (33.8)	94 (39.7)
Self-inflicted stress	12.23 (4.22)	49 (20.7)	113 (47.7)	75 (31.6)

**Figure 1:**
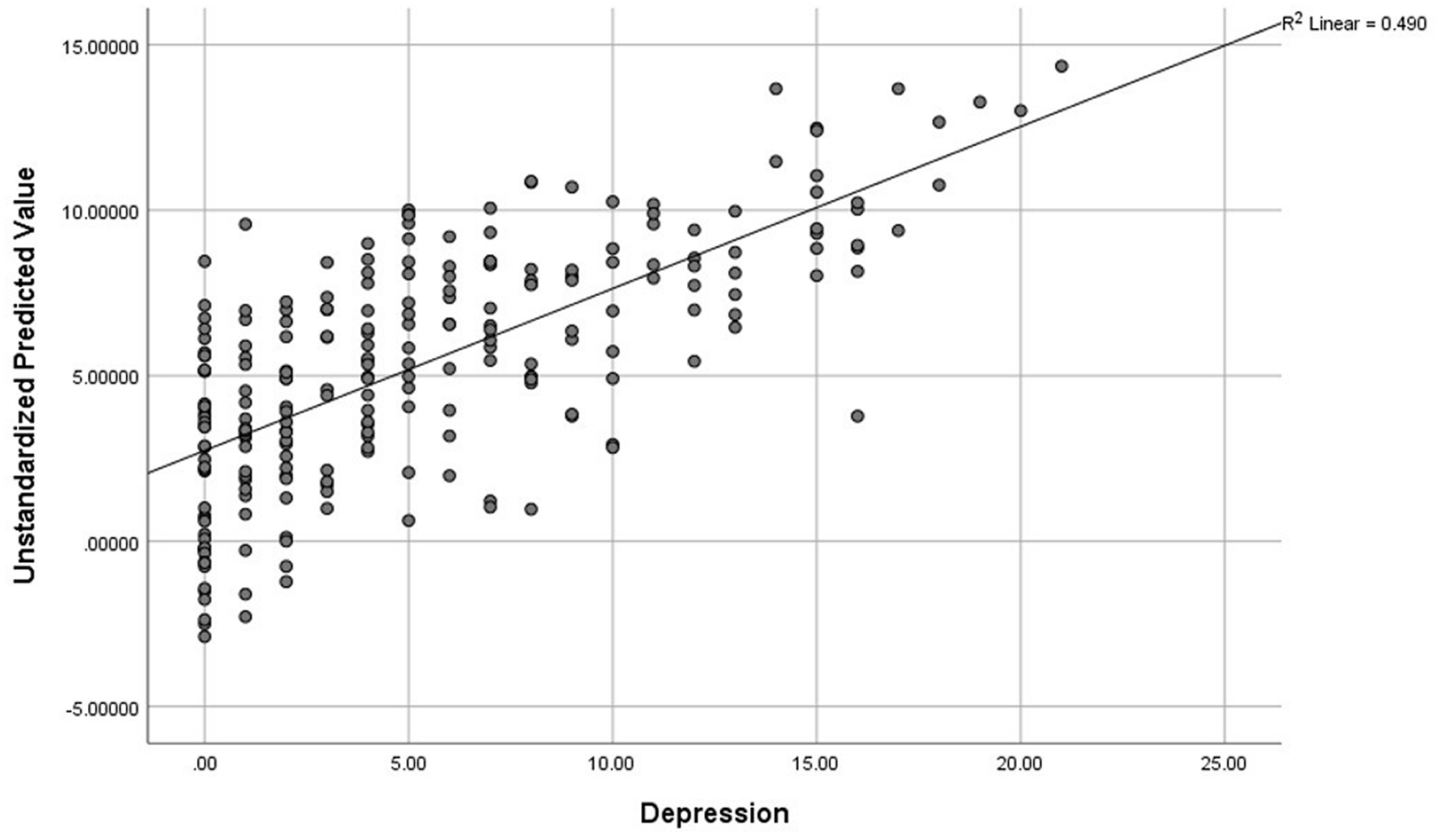
A scatterplot of the relationship between the predictive variables and depression for the regression model.

Table 4 demonstrates statistically significant positive correlations between all academic stress subscales and depression (*p* < 0.001). Table 5 illustrates that study year (*β* = 0. 0.254, *p* = 0.03), sleep problem (*β* = 0.165, *p* = 0.002), studying in group stress (*β* = 0.347, *p* < 0.001), and self-inflected stress (*β* = 0.206, *p* = 0.020) are the significant predictors of depression. Studying in group stress was the main significant predictor of depression which accounted for 34.7% of the variance. The regression model explains 49.0% of the variance in the depression scale (*F* = 17.916, *p* < 0.001). Figure 1 also shows the scatterplot of the relationship between the predictive variables and the depression. It explained variance of depression for the regression model where the dots are much tighter around the line of fit. In addition, the post-hoc comparison was conducted and indicated that the mean score of depression was significantly different for first year students from second, third, and fourth year (*F* = 5.787, *p* = 0.001).

**Table 4:** Correlation between academic stress and depression (*n* = 237).

		Depression	Teachers’ Stress	Results Stress	Test stress	Studying in group stress	Peer stress	Time-Management stress	Self-Inflicted stress
Depression	*r*								
	*p*								
Teachers’ stress	*r*	0.464^*^							
	*p*	<0.001							
Results stress	*r*	0.378^*^	0.550^*^						
	*p*	<0.001	<0.001						
Test stress	*r*	0.387^*^	0.664^*^	0.661^*^					
	*p*	<0.001	<0.001	<0.001					
Studying in group stress	*r*	0.601^*^	0.516^*^	0.456^*^	0.527^*^				
	*p*	<0.001	<0.001	<0.001	<0.001				
Peer stress	*r*	0.449^*^	0.556^*^	0.543^*^	0.559^*^	0.633^*^			
	*p*	<0.001	<0.001	<0.001	<0.001	<0.001			
Time-management stress	*r*	0.505^*^	0.649^*^	0.554^*^	0.672^*^	0.644^*^	0.576^*^		
	*p*	<0.001	<0.001	<0.001	<0.001	<0.001	<0.001		
Self-inflicted stress	*r*	0.538^*^	0.712^*^	0.669^*^	0.713^*^	0.597^*^	0.584^*^	0.704^*^	
	*p*	<0.001	<0.001	<0.001	<0.001	<0.001	<0.001	<0.001	

*r*, Pearson coefficient;*Statistically significant at *p* ≤ 0.001.

**Table 5:** Multiple linear regression analysis showing the effect of different factors on depression (*n* = 237).

**Variable**	***B***	**Standardized coefficients beta**	***t***	***p***	**95% CI**	
					**LL**	**UL**
Age	−0.320	−0.090	−1.095	0.275	−0.895	0.256
GPA	−0.171	−0.024	−0.481	0.631	−0.874	0.531
Study year	1.149	0.254	2.413	0.003*	0.394	1.904
Residence	−1.569	−0.082	−1.685	0.093	−3.403	0.266
Sleep problem	1.676	0.165	3.088	0.002*	−0.607	2.764
Teachers’ stress	0.051	0.080	1.076	0.283	−0.042	0.143
Results stress	0.064	0.065	0.906	0.366	−0.075	0.202
Test stress	−0.100	−0.090	−1.080	0.281	−0.284	0.084
Studying in group stress	0.344	0.347	4.873	<0.001*	0.205	0.484
Peer stress	0.032	0.024	0.340	0.734	−0.153	0.216
Time-management stress	0.066	0.046	0.584	0.560	−0.156	0.288
Self-inflicted stress	0.248	0.206	2.345	0.020*	0.040	0.457
*R*^2^ = 0.490, *F* = 17.916, *p* < 0.001						

*F*, *p*, f and *p* values for the model.

*R*^2^, coefficient of determination.

*B*, unstandardized coefficients.

Beta, standardized coefficients.

*t*, t-test of significance.

LL, lower limit; UL, upper limit.

## 4 Discussion

This study aimed at examining the perceived level of depression and academic stress as well as examining the associations between the variables among nursing students. Based on the results, nursing students perceived normal to mild levels of depressive symptoms, which is contrary to the study that was conducted by Soltan et al. (40). Soltan et al. revealed that most medical students were presented with severe levels of depression symptoms. These differences could be attributed to the time of data collection. Most previous studies were implemented during COVID-19. The factor that could be attributed to the lower levels of depressive symptoms in this study is the availability of strong support system in the College of Nursing, family, and friends. Student Supervisors and Social Specialists are available for all students in the College of Nursing. The Social Specialists are responsible for negotiating students’ issues, providing counseling services, and referring students with problematic issues internally and/or externally to specialty clinics. Also, the availability of strong social support from family as reported by most of the participants who live with their family could be an indicator for the low level of depression score.

This study revealed that the mean scores for the academic stress subscales were found to be moderate in almost more than half of the participants, except for test stress and time-management stress scores which were high. Along with previous studies, Joseph et al. (41) revealed that 77.3% of the participants reported moderate level of academic stress. Also, most students in Alhamed’s (42) study perceived mild to moderate academic stress levels while 8.6% perceived severe levels of academic stress. In line with previous study that was conducted by Facioli et al. (7), the highest means of academic stress were test stress and time-management stress. These similarities could be attributed to the rigorous of nursing curricula, the pressure to perform well in the exams, fear of failure, and lack of time-management skills among nursing students.

All academic stress subscales were correlated significantly with depression. Along with the previous study that was conducted by Deng et al. (43), academic stress and depression were significantly correlated with a positive relationship. They also indicated that academic stress can lead to depression and negatively affect students’ performance and learning outcomes among students. The multiple regression showed that studying in group stress was the main significant predictor of depression among the study participants. Individuals may have group conflicts, difficulties in coordinating schedules, and difficulties maintaining focused when they study in groups. In addition, students in group study may share challenging concepts, have individual differences and preferences, or compare their progress and expectations, which could potentially intensify the risk of depression.

Another predictor of depression in this study was the self-inflicted stress. This relationship was observed in a previous study that was conducted by Menon et al. (44). Self-inflicted stress among students often arises from setting high expectations for academic performance or meeting unrealistic academic goals. Students may constantly strive for high achievements and feel intense pressure to meet their own standards. High risk of depression may arise among this population from continuous self-criticism and self-doubt of their abilities, unfavorably comparing themselves to their peers, and feeling of hopelessness to achieve high academic performance (44).

The regression model depicted that study years is another significant predictor of depression. The post-hoc analysis showed that first-year students reported a statistically significant high level of depression compared to second, third, and fourth-year nursing students. Janatolmakan et al. (22), Kokou-Kpolou et al. (45), Kwak et al. (11), Nakalema and Ssenyonga (46), and Tuffah and Al-Jubouri (47) reported the same finding, while Bamuhair et al. (48) and Ngasa et al. (49) reported that students had high depressive symptoms in the final year of study. The variations between the results could be attributed to unfamiliarity of the first-year students with management skills, educational environment, academic procedures and requirements, lack of adaptability to cope with the new circumstances, high expectations from family and faculty, and concern over failure.

The multiple regression also revealed that sleep problem was a predictor of depression. In line with previous study, Belingheri et al. (50) found that chronic insomnia was significantly correlated with mood disorders, particularly depression. Staying up late before all the big and small school tests could attribute to this result.

### 4.1 Implications of the study

The burden of academic life and stressful academic circumstances as well as poor coping strategies among students to deal with difficulties in the academic setting increase their vulnerability to depression. Identification of stressors and predictors of depression becomes essential to prevent the development of negative effects on academic performance, psychological distress, and emotional exhaustion (51).

### 4.2 Recommendations

Several strategies can be used for managing stress while studying in a group such as establishing open communication between group members to express their concerns, preferences, and expectations, and to freely share their thoughts, questions, and insights (52). Mutual respect should be fostered between group members where everyone’s ideas and diverse viewpoints are respectfully heard and considered. Instructors can facilitate group formation by conducting icebreaker activities or team-building exercises which help students get to know each other. They can provide guidelines and resources for effective group work strategies and communication skills. Instructors can also provide guidance on conflict resolution strategies and encourage students to actively listen to each other and respect diverse perspectives (52).

Understanding the cause of self-inflicted stress is important for successfully managing the stress. Students with high self-inflected stress should be aware of their own capacity and limitations and seek help to deal with stress. A supportive network of individuals who provide help when needed and limiting exposure to negative influences and stressors are crucial for managing self-inflicted stress. To manage sleep problems and improve sleep quality, students should maintain a consistent sleep schedule, create a comfortable sleep environment, avoid stimulating activities before bedtime, and limit long or frequent naps that could disrupt the sleep schedule (53).

To reduce academic stress and depression, participants must be aware of coping strategies to deal with academic expectations and demanding situations. Attention should be paid on teaching the students about time management, stress management, and problem-solving strategies to improve their self-esteem. Students must be educated about the thoughtful use of time available, and how to set goals and prioritize activities to study for their examinations and complete their task.

Proper counseling sessions to deal with the anticipatory suffering and timely academic consultation should be activated. The counselors must help students to express their emotions, and plan beforehand to prevent depression from occurring. Interactive academic sessions on stress management can encourage nursing students to express their issues and emotions, plan problem solving, meeting the academic students’ needs, and even taking self-responsibility to single out each problematic issue.

Other common coping strategies that used by students were attending extracurricular activities, resilience training programs, mindfulness-based therapy, physical activity, and meditation classes, going out with friends, and getting adequate sleep (41, 54). Ruzhenkov et al. (55) indicated that psychotherapy sessions and training programs that improve social intelligence and reduce emotional tension should be offered by every institution.

Undergraduate students especially at this age need support from family, friends, peers, and faculty members. Faculty members must play a constructive role in guiding and mentoring students and must be responsible to make a healthy and stress-free educational environment. They can also alleviate the test stress and result stress by providing frequent mock examinations. Developing an individualized mental health intervention program that addresses academic stress and problematic issues is necessary to control depression and academic stress among nursing students. Continuous observation and follow-up with the intervention programs and including these interventions in the curriculum on a consistent basis are equally important (11). Greater institutional support must be emphasized for the need of more collaborative actions especially for students who are vulnerable to higher levels of academic stress and depression (40).

### 4.3 Limitations of the study

This study was implemented on female nurses only and in one of the nursing colleges, therefore the findings cannot be generalized to this population. More studies need to be implemented in other regions to better understand how academic stress is associated with depression and students’ performance. Another limitation is that data was collected by using a self-reported survey that was filled out by students. This could affect the results of the study by imposing response bias. Further, the cross-sectional study design possesses a limitation of testing the causality. It is necessary to investigate the study variables using longitudinal study design in future research. In addition, further studies are needed to examine other factors that may have direct or indirect associations with depression such as quality of life, satisfaction with the quality of teaching, and support from instructors, family, and peers.

## 5 Conclusion

This study aimed at examining the perceived level of depression and academic stress and the association between depression and academic stress among nursing students in Saudi Arabia. About 57% of the participants perceived normal to mild level of depression and between 47.7 and 56.1% of them perceived moderate levels of academic stress subscales, except time management stress and test stress subscales that were reported as high levels (46 and 39.7%, respectively). All academic stress subscales had a statistically significant positive correlation with depression. The main significant predictor of depression among the study participants is studying in group stress, followed by self-inflected stress, study year, and sleep problem. Open communication and mutual respect should be fostered between group members. Facilitation of group formation and teaching students about communication skills should be prioritized to manage group stress. Understanding the cause of self-inflicted stress and one’s own capacity and limitations and limiting exposure to negative influences are imperative to reduce self-inflected stress. Maintaining a comfortable sleep environment and a consistent sleep schedule, while avoiding stimulating activities before bedtime will enhance the quality of sleep. Students need to be educated about time-management, problem-solving, negotiation, and coping strategies to deal with academic expectations and demanding situations. They must seek support when needed, especially during their first year of education.

## Data availability statement

The datasets generated and/or analyzed during the current study are not publicly available due to data privacy but are available from the corresponding author on reasonable request.

## Ethics statement

The studies involving humans were approved by the Institutional Review Board, Princess Nourah bint Abdulrahman University, approval (No. 22–0488). The studies were conducted in accordance with the local legislation and institutional requirements. The participants provided their written informed consent to participate in this study.

## Author contributions

SA: Writing – review & editing, Writing – original draft, Visualization, Validation, Supervision, Software, Resources, Project administration, Methodology, Investigation, Funding acquisition, Formal analysis, Data curation, Conceptualization.
